# Investigation of possible G-quadruplex formation by GU- and GA-rich repeats and their role in translation

**DOI:** 10.1080/15476286.2025.2512613

**Published:** 2025-06-02

**Authors:** Bas M. Morren, Jitske Marcelis, Iza Muradin, René C.L. Olsthoorn

**Affiliations:** Leiden Institute of Chemistry, Leiden University, Leiden, The Netherlands

**Keywords:** G-quadruplex, dinucleotide repeats, ribosomal frameshifting, translation, PhenDC3

## Abstract

RNA G-quadruplexes (rG4s) are involved in many aspects of cellular and viral protein expression. rG4s consist of at least two stacks of guanine tetrads that are stabilized by non-Watson-Crick-Franklin base pairs. It is currently unknown how single or multiple non-G nucleotide insertions affect the stability or function of rG4s. Here, we investigated the G4-forming potential of GU- and GA-rich sequences by measuring their ability to inhibit ribosomal scanning and induce −1 ribosomal frameshifting (−1 FS) using a cell-free lysate. Our results show that, in contrast to canonical rG4s, GU and GA repeats with eight or more guanines do not affect ribosomal scanning or stimulate −1 FS. However, in the presence of G4-stabilizing ligands PhenDC3 or pyridostatin, GU and GA repeats strongly inhibited scanning and induced −1 FS. These findings have implications for the structural landscape of rG4s and the potential side-effects of G4 targeting drugs in general.

## Introduction

Gene expression is a complex process that is tightly regulated at multiple levels to ensure precise control of protein synthesis. Although transcriptional regulation has been extensively studied, post-transcriptional regulation has emerged as a critical mechanism for fine-tuning gene expression. This process relies mainly on RNA-binding proteins that recognize specific structural elements in mRNAs. One such element is the G-quadruplex (G4), a structure that can form in guanine-rich regions in both DNA and RNA [1,2] and that consists of stacks of guanine tetrads that are stabilized by non-Watson-Crick-Franklin (WCF) base pairs. While the role of these structures is relatively well studied in DNA, RNA G4s (rG4s) have only been the focus of research more recently. rG4s have now been found in mRNAs, non-coding RNAs and viral RNAs [3] and can also form intermolecularly from tRNA fragments [4].

rG4s have been shown to play a role in pre-mRNA splicing [5–7], gene silencing [8–10], alternative polyadenylation [11], RNA localization [12], translation regulation [13] and RNA stability [14]. In mRNA, rG4s have been found to be highly enriched in the 5’ and 3’UTRs, where they can regulate translation. In the 5’UTR, rG4s mainly lower translation by blocking ribosomal scanning, but positive regulation has also been described [4,15–17]. Other findings suggest that rG4 formation could compete with Repeat Associated Non-AUG translation (RAN), where the sequence GGGGCC, for example, either is available for binding of translation factors or forms G4s [18,19]. Within coding regions, the presence of rG4s has been suggested to play a role during translation elongation, affecting the folding of nascent proteins [20,21] and to stimulate −1 ribosomal frameshifting (−1 FS) [22].

In addition, rG4s have been discovered in several viruses, including Zika virus and SARS-CoV-2 [23–26]. For these and other viruses, G4 stabilizing ligands have been shown effective as potential anti-viral compounds [8,27–31]. Similarly, G4 targeting ligands have also gained interest as a way to combat antibiotic resistant bacteria [32–35]. For example, in *Mycobacterium tuberculosis*, the stabilization of putative rG4s in PE/PPE genes caused the selective suppression of growth and possibly pathogenicity [36]. Last but not least, G4s have also shown to be promising targets for anti-cancer drugs [37,38].

Much of our fundamental understanding of G4 structures in RNA, however, is still lacking. For DNA, it has been well described that G4s can adopt a large variety of conformations, including parallel, anti-parallel, and hybrid structures. In addition, possible variations away from the canonical G4 sequence are far better documented for DNA, for example, *c-KIT* and *c-MYC* promoter G4s include one or more bulged nucleotides in one of their G-stretches [1]. The number of well-characterized RNA G4s, however, is currently quite limited, but the solved structures of several RNA aptamers indicate that also in RNA a large variety in topology is possible [39].

Currently, several algorithms are available for predicting G4s in DNA and RNA. Many of these are based on the assumption that two or more stacks of G-tetrads are required to adopt a G4 structure and that longer loop lengths lead to progressively lower G4 stability. However, the examples of *c-KIT* and *c-MYC* indicate that guanines can be interrupted by one or more other nucleotides. Many of the newer self-learning algorithms also lack properly validated datasets to train themselves on [40–42] and reviewed in [43,44]. To address the conformational landscape of RNA G4s we here investigated several potential G4 forming sequences that contain one or more bulges. As a measure of G4 formation of these sequences, we tested their ability to impede ribosomal scanning and to stimulate −1 FS in cell-free lysates. Our results suggest that depending on the presence of certain ligands or ions the topology of certain sequences can be dramatically altered, to the point that even sequences far from the canonical rG4 consensus sequence can be forced into an rG4 conformation. The implications of these findings for our knowledge of the rG4 landscape and potential side-effects of G4 targeting drugs are discussed.

## Material and methods

### Plasmid construction

Complementary DNA oligonucleotides (SigmaAldrich) were annealed by heating a mixture containing 44 μl Milli-Q water, 5 μl Green buffer (Thermo Fisher), 0.5 μl of both oligonucleotides (100 mm), to 100°C for 15 min and then allowed it to cool down to RT. These oligonucleotides were designed to have, after annealing, the required overhangs to clone them into the KspAI and Van91I restriction sites upstream of the Renilla luciferase gene of plasmid pMRL [45]. For non-radioactive frameshifting assays, a pDual plasmid [46] was digested with Acc65I and BamHI (New England Biolabs), and annealed oligonucleotides containing the putative G4 sequence and slippery site were inserted in between the firefly and Renilla luciferase genes []. All ligations were performed at 16°C overnight with T4 DNA ligase, according to manufacturer’s specifications (Thermofisher). For radioactive frameshifting assays, a variant of the pSF plasmid containing a UUUAAAC slippery sequence [47] was used to insert complementary oligonucleotides into the Acc65I and NcoI sites. After ligation, the plasmids were used to transform E. coli XL10 cells and plasmid DNA was isolated using the PureYield^TM^ Plasmid Miniprep System (Promega). All constructs were verified by DNA sequencing (LGTC, Leiden, The Netherlands). A list of the oligonucleotides used for cloning is shown in Supplementary Tables S1 and S2.

### Preparation of RNA

Plasmids were linearized with either XhoI (pMRL and pDual) or BamHI (pSF), followed by concentration of the template by ethanol precipitation. A total of 125 ng of digested plasmid was used as a template for 5-μl transcription reactions, otherwise following manufacture’s specifications (Promega and New England Biolabs). For the pSF constructs, an SP6-transcription kit was used (Promega and New England Biolabs). After transcription, the RNA was checked and quantified by 1% agarose gel electrophoresis and diluted to the desired concentration.

### In vitro translation for translation efficiency

Translation mixtures contained 2.5 μl nuclease-treated rabbit reticulocyte lysate (Promega), 0.25 μl amino acid mixture minus cysteine, 0.25 μl amino acid mixture minus methionine, 50–100 ng RNA and the indicated amounts of KAc, NaAc, PhenDC3 or pyridostatin (SigmaAldrich), to a total volume of 5 μl. After an incubation for 1 h at 28°C, the reaction was stopped by adding 45 μl of 10 mm Tris (pH 7.5) buffer. Twenty microlitres of this mixture was transferred to a 96-well plate, and luciferase activity was measured after the addition of 2.5 μl diluted Renilla-Glo Luciferase Assay Substrate (Promega) in a GloMax-multi luminometer. The data of these assays were, per construct, normalized to the signal received from samples without added ions, PhenDC3 or pyridostatin. Significance of differences compared to the negative control were tested with a two tailed equal variance t-test.

### In vitro translation for frameshifting

The luminescent frameshifting assay was also performed in rabbit reticulocyte lysate as described above: however, 20 μl each of the stopped reaction was transferred to two different wells, one used to measure the *Renilla* luciferase signal as above, and the other to measure the firefly luciferase signal. This was done by adding 2.5 μl of firefly luciferase substrate (Promega) and measuring the signal as above. The data from these assays were analysed by first dividing the firefly signal over the *Renilla* and then comparing this ratio to an in-frame control under the same conditions.

In radioactive frameshifting assays 0.5–1 μl of EasyTag™ EXPRESS35S Protein Labelling Mix (PerkinElmer), which is an amino acid mixture containing both ^35^S-L-methionine and ^35^S-L-cysteine (>11 mCi/mL), was used in the 5-μl translation mixture as described above. After 1 h incubation at 28°C, the reaction was terminated using 5 μl of 2× Laemmli sample buffer (Thermo Fisher). After boiling the samples for 5 min, four microlitres of the sample were analysed on 12.5% SDS polyacrylamide gels and dried. Once dry, the gels were exposed to a phosphor imager plate (Molecular Dynamics) for 2–4 days. The plate was then imaged using a Typhoon™ BioMolecular Imager (GE Healthcare). The frameshifting percentage was calculated using Quantity One software (Bio-Rad) by measuring the intensity of the frameshifted and non-frameshifted products, correcting for the number of cysteines + methionines and determining the ratio between them. All constructs were tested at least twice.

### Circular dichroism (CD)

For the acquisition of the CD spectra a Jasco J-810 spectropolarimeter was used, equipped with a Peltier temperature controller. All spectra were measured at 5°C and at a scanning rate of 100 nm/min, within the wavelength range of 210–320 nm. The samples were prepared in 10 mm Tris (pH 7.5) buffer and contained 2–5 μM RNA and were measured in 10 mm path length quartz cuvettes. After recording the spectra, the background spectrum was subtracted, and the curve was smoothed using the Savitzky-Golay algorithm at a convolution width of 13. Finally, the ellipticity at 320 nm was set to zero. The measured molar ellipticity was corrected for the concentration of RNA and the cuvette size using the following formula: [θ] = 100·θ_obs_ /(C·L), where [θ] is the molar ellipticity (deg·cm^2^·dmol^−1^), θ_obs_ is the observed ellipticity (mdeg), C is the concentration (mol/l), and L is the cell path length (cm).

## Results

In this study, an in vitro translation assay was used to quantify the expression of Renilla luciferase mRNAs with various 5’UTR inserts. First, the assay’s sensitivity was evaluated by measuring the translational efficiency (TE) of two canonical RNA G-quadruplexes: (GGGU)_4_ and (GGU)_4_. (GGGU)_4_ is anticipated to adopt a more stable G4 because of the additional G-tetrad it can form in the presence of various alkali metal ions, which have been reported to stabilize G4s [48]. It is important to note that the lysate already contains potassium and sodium ions, and the concentrations mentioned are those of the added cations. Two known G4 stabilizers, PhenDC3 (DC3) and pyridostatin (PDS), were used to investigate whether G4s could be further stabilized. A construct incapable of forming a G4 was used as a negative control (N.C.).

**Figure 1.**
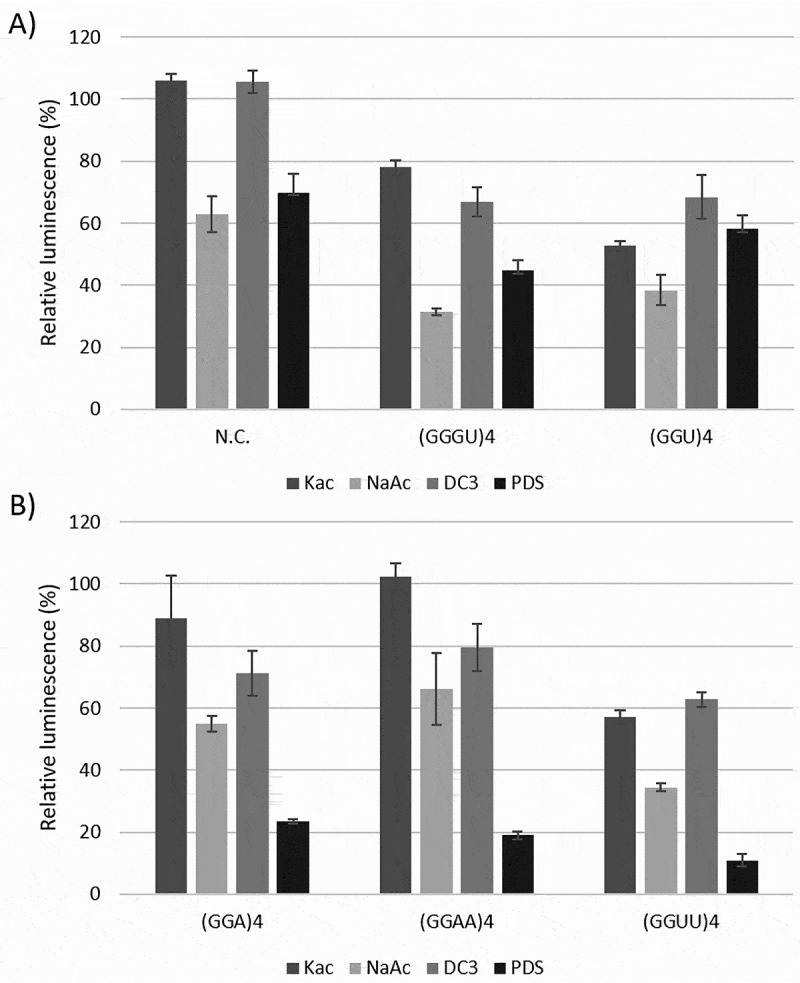
Effect of salts and ligands on the translation efficiency of luciferase mRNAs containing various rG4s in their 5′UTR. A. Relative luminescence of the negative control (N.C.), (GGGU)_4_, and (GGU)_4_ constructs and B. of (GGA)_4_, (GGAA)_4,_ and (GGUU)_4_ constructs in the presence of added 150 mm KAc, 100 mm NaAc, 1 μM PhenDC3 (DC3) or 2 μM pyridostatin (PDS). Note that the potassium ion concentration introduced by the lysate is 56 mm and raises [KAc] to a final 206 mm (36). All assays were conducted at least twice in duplicate and were normalized to a control containing no additive. Shown here is the average of the results obtained with the average standard deviation. Significance is shown for differences compared to the N.C. in similar conditions (ns = p-value >0.05, * = p-value ≤0.05, ** = p-value ≤0.01, *** = p-value ≤0.001). The exact sequences of the constructs are listed in Supplementary table S1.

The addition of 150 mm potassium acetate had a minimal impact on the overall TE of N.C., whereas the TE of G4-containing constructs dropped to approximately 78% and 53% for three- and two-stack G4s, respectively (Figure 1(A)). The smaller decrease observed for (GGGU)_4_ can be attributed to its inherently higher stability compared to that of (GGU)_4_ resulting in a lower TE owing the presence of potassium ions in the lysate [49]. It should be noted that potassium is known to stimulate translation. Indeed, when exchanging half of the lysate in the reaction with a buffer not containing potassium the TE of (GGGU)_4_ initially increased to 200% before falling, when increasing amounts of potassium were reintroduced, whereas N.C. also increased but remained steady at higher concentrations (see Supplementary Figure S1). Sodium acetate reduced the TE of the two G4 constructs to 31% and 38%. However, as the TE of N.C. was reduced to approximately 63%, the reduction observed for (GGGU)_4_ and (GGU)_4_ was not solely due to G4 formation but also due to the known adverse effect of sodium ions on translation [50]. Nonetheless, both salts appeared to have a more significant impact on the G4-containing constructs than on the control, suggesting that potassium and sodium can stabilize these rG4s. In addition to N.C., a firefly luciferase control mRNA not capable of forming an rG4 was also used to verify the general effects of salts on translation. The effects for all conditions were nearly identical to those of N.C. (Supplementary Figure S2 and Table S3).

For the addition of PhenDC3 and PDS, as with the salts. PhenDC3 affected both (GGGU)_4_ and (GGU)_4_ equally (TE reduction to ~68%), and PDS lowered their TEs to approximately 45% for (GGGU)_4_ and 58% for (GGU)_4_. PhenDC3 did not affect the translation of N.C., whereas PDS reduced the TE to 70%. Altogether, both ligands seem to have similar effects on stabilizing either (GGGU)_4_ or (GGU)_4_ when taking into account the effect of PDS on the negative control.

To determine if we could replicate this reduction pattern for a slightly different G4, the (GGA)_4_ sequence was tested. This construct showed a TE similar to that of the negative control for both potassium and sodium, indicating that the addition of ions did not induce a G4 in this construct, at least not one that is stable enough to impede ribosomal scanning. The addition of PhenDC3 lowered TE to 71% (Figure 1(B)), suggesting that a stable G4 can be formed when a G4 specific ligand is added. PDS also showed this more clearly, as indicated by the large reduction in TE (to ~24%).

In the next set of experiments, we analysed less stable G4s by doubling the loop size between the G-stretches [51]: (GGUU)_4_ and (GGAA)_4_. Comparing these constructs to the respective single-nucleotide loop variants, it is striking to see similar reductions in TE. For (GGU)_4_ compared to (GGUU)_4_ we observe that the effect of both salts and the addition of PhenDC3 show similar effects despite the longer loops in (GGUU)_4_ (Figure 1(B)) [51,52]. Interestingly, the addition of PDS resulted in a far greater reduction in TE for (GGUU)_4_, dropping it to 11%. The effect on translation of (GGA)_4_ and (GGAA)_4_ on the other hand is fairly identical. Both had their translation less affected by the addition of salts, slightly reduced by PhenDC3, and strongly decreased by PDS. These results suggest that (GGAA)_4_ does not adopt a (stable) G4 in the presence of salts and can only form one with the aid of PhenDC3 or PDS. Interestingly, while PDS for the uracil-containing G4s showed a clear difference when considering loop length, this was not observed for (GGA)_4_ and (GGAA)_4_. PhenDC3 and both salts showed far less reduction in TE for the adenosine-containing G4s, to a point at which it becomes indistinguishable from the negative control. PhenDC3’s lesser effectiveness compared with PDS may be due to a different binding mode and/or preference for certain loop nucleotides, for example, A or U.

### Interrupted stretches of eight or more Gs can be forced into a G-quadruplex

**Figure 2.**
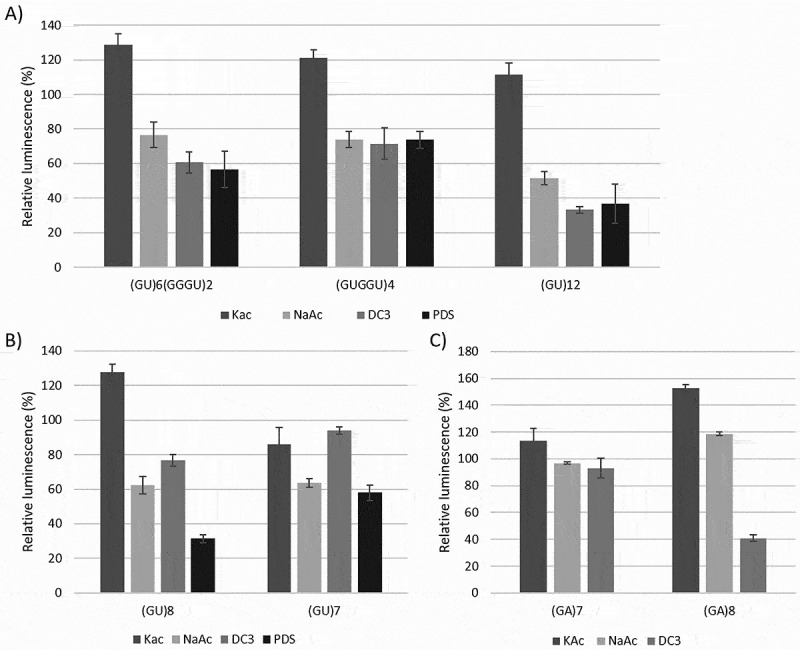
Effect of salts and ligands on the translation efficiency of luciferase mRNAs containing various putative rG4s in their 5’ UTR. A. Relative luminescence of (GU)_6_(GGGU)_2_, (GUGGU)_4,_ and (GU)_12_ constructs, B. Relative luminescence of (GU)_8_ and (GU)_7_ constructs, and C. Relative luminescence of (GA)_7_ and (GA)_8_ constructs in the presence of added 150 mm KAc, 100 mm NaAc, 1 μM PhenDC3 (DC3) or 2 μM pyridostatin (PDS). See legend to Figure 1 for further details.

After establishing that our assay was capable of monitoring G4 formation, we investigated to what extent other G-rich sequences would affect translation. By inserting different numbers of uracils between the guanines, the following three constructs were made: (GU)_6_(GGGU)_2_, (GUGGU)_4_ and (GU)_12_. Interestingly, the addition of potassium acetate in these cases resulted in a slight increase in TE of up to 129%. Adding sodium acetate to (GU)_6_(GGGU)_2_ and (GUGGU)_4_ resulted in a TE that was slightly above that of the negative control, 76% and 74%, respectively (Figure 2(A)), while (GU)_12_ performed slightly worse than the negative control (52%); however, these changes were not statistically significant. The addition of PhenDC3 and PDS, however, had large effects on translation; the TE of (GU)_6_(GGGU)_2_ and (GUGGU)_4_ dropped to 55–60% and 70–75%, respectively (for both ligands), and that of (GU)_12_ even decreased to 33%. These results suggest that all three constructs can be forced to adopt a quadruplex made up of three guanine tetrads with the intervening uracils presumably bulging out, or forming U-tetrads, or both (compare Figure 5(A–C)). Shorter variants of the (GU)_12_ repeat showed that translation of (GU)_8_ was strongly reduced by the addition of PhenDC3 and PDS, while that of (GU)_7_ was not. These results suggest that (GU)_8_ is able to form a G4 composed of just two G-tetrads, while seven guanines are not sufficient to form a G4 (Figure 2(B)). This was also confirmed using (GA)_7_ and (GA)_8_ constructs: the addition of PhenDC3 had, essentially, no effect on (GA)_7_ translation but reduced translation of (GA)_8_ to 41% (Figure 2(C)), whereas the addition of potassium and sodium had a neutral or positive effect on the translation of both constructs. From this, we conclude that PhenDC3 can also induce a G4 conformation in GA repeats, provided that at least eight guanines are present.

### GU and GA repeats stimulate ribosomal frameshifting in the presence of PhenDC3

**Figure 3.**
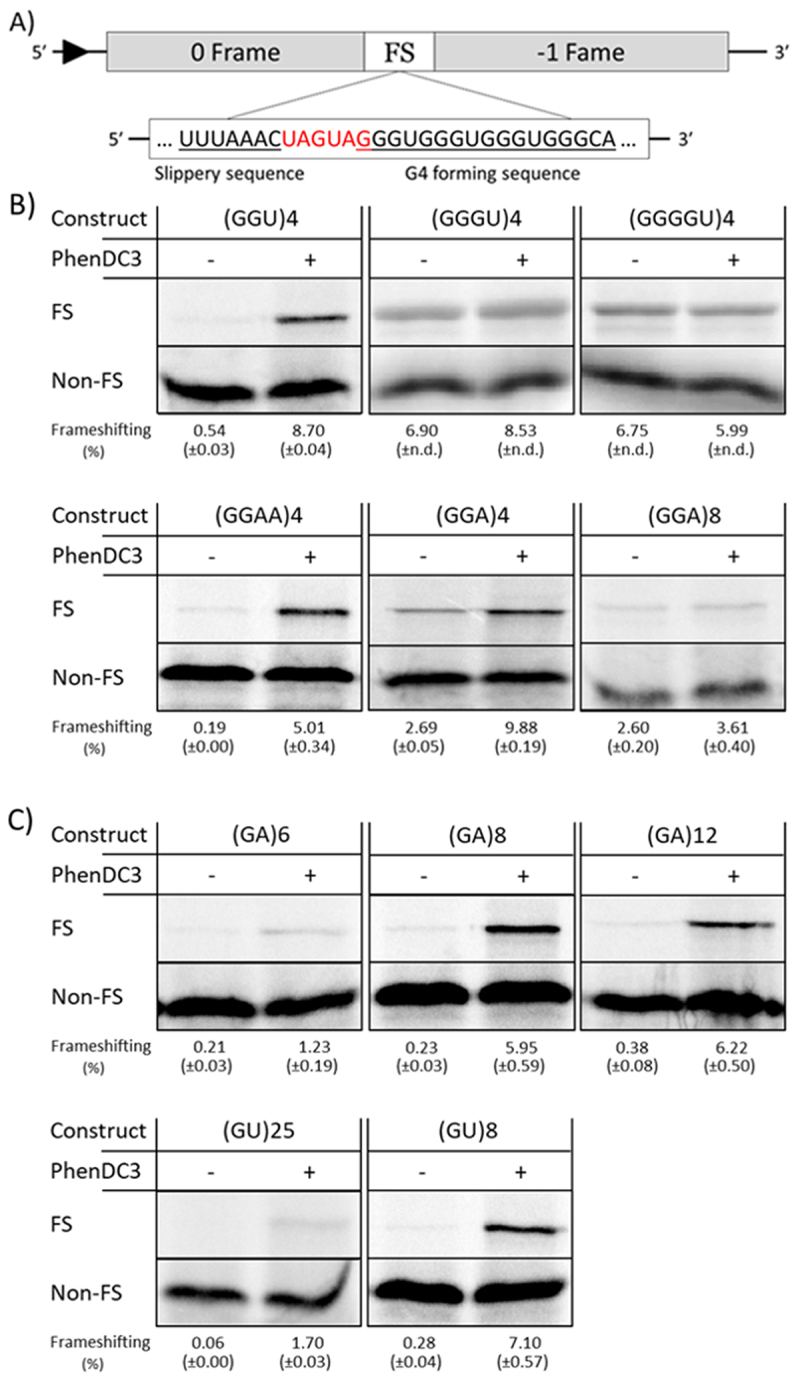
Analysis of − 1 FS stimulation by canonical G4s and GU and GA repeat containing constructs, with and without the addition of PhenDC3 (DC3). A) Schematic view of pSF frameshift-reporter plasmid. The protein in the 0 frame is terminated just after the (UUUAAAC) slippery site unless the G4 forming sequence (here (GGGU)_4_) is capable of inducing − 1 frameshifting. The G4-forming sequence is exchanged for the indicated sequences with the addition of 1–2 nucleotides to ensure the second open reading frame is in the − 1 frame. B) Autoradiogram showing ^35^S-labelled translation products of the indicated constructs in RRL. −1 FS is monitored by the presence of a 65-kD product, indicated by ‘FS’. The 0-frame product is indicated by ‘non-FS’. Quantitative analysis of frameshifting efficiency (%) is described in ‘materials and methods’ section. The standard deviation (S.D.) is derived from at least two independent experiments. C) autoradiogram for GA and GU repeats containing constructs. Unprocessed autoradiograms are shown in Supplementary figure S4. The exact sequences used are listed in Supplementary table S2.

Previously, it was shown that canonical rG4s can stimulate −1 ribosomal frameshifting [51,54]. As an additional measure of G4 formation, the capacity of the GU and GA repeats to stimulate −1 FS was also investigated. For this purpose, a variant of the pSF plasmid containing a UUUAAAC slippery sequence [] was used which produces a short protein in the 0 frame and a longer protein when ribosomes shift into the −1 frame (Figure 3(A)).

The canonical G4-forming sequences (GGGU)_4_ and (GGGGU)_4_ caused 4.5% and 5.5% of ribosomes, respectively, to shift into the −1 frame, comparable to previously reported levels of −1 FS [22]. The addition of PhenDC3 raised this percentage to ~6.5 for both constructs, indicating that (GGGU)_4_ and (GGGGU)_4_ are sufficiently stable on their own to induce ribosomal frameshifting. The (GGU)_4_ construct showed merely 0.54% of −1 FS, about two times above background level, whereas (GGA)_4_ induced 2.7% (Figure 3(B)), suggesting that the latter forms a more stable G4 than (GGU)_4_. Interestingly, in the presence of PhenDC3–1 FS levels increased to 8.7% and 9.9%, respectively, thereby exceeding those of the (GGGU)_4_ and (GGGGU)_4_ constructs. A similar effect was observed for (GGAA)_4_; 0.19% of −1 FS in the absence of PhenDC3 but 5.0% in the presence of PhenDC3, suggesting that (GGAA)_4_ by itself does not form a G4 of sufficient stability to stimulate −1 FS.

Next, we investigated the ability of the GA- and GU-rich repeats to induce frameshifting. As shown in Figure 3(C), (GA)_6_, (GA)_8_, (GA)_12_, (GU)_25_ and (GU)_8_ were unable to induce FS above background levels (0.21%, 0.23%, 0.38%, 0.06% and 0.28% respectively). However, the addition of PhenDC3 significantly increased frameshifting by (GA)_8_, (GA)_12,_ and (GU)_8,_ raising FSE to 5.95%, 6.22%, and 7.10%, respectively, while the longer (GU)_25_ displayed a low FSE (1.70%) possibly due to the fact that many G4s could be formed further away from the slippery sequence. (GA)_6_, which is too short to form a G4 without incorporating guanine residues from further upstream or downstream regions, exhibited a low frameshift efficiency (FSE) of 1.23%. These data strongly suggest that GA and GU repeats form G4-like structures only in the presence of PhenDC3.

A bicistronic reporter containing firefly and Renilla luciferase was also used to investigate frameshifting of (GU)_8_ and (GGU)_4_. The addition of KAc had no effect on the FSE of (GU)_8_ or of (UA)_8_ which served as a negative control but led to an increase in FSE of (GGU)_4_ indicating that its G4 conformation was stabilized (Supplementary Figure S3). The addition of PhenDC3, however, strongly enhanced FSE of both (GU)_8_ and (GGU)_4_, while no significant effect was observed for the UA repeat, again indicating that PhenDC3 can induce a G4 in (GU)_8_.

### CD analysis of GU and GA repeats

The above results suggest that the G4 specific ligands PhenDC3 and PDS are capable of forcing G-rich sequences into functionally stable G4s. However, we were interested to find out the native structure of the GA and GU repeats and the effect of salts thereon. To this end, circular dichroism (CD) spectroscopy [55,56] was performed with GA- and GU-rich oligoribonucleotides in Tris buffer, with or without additional potassium chloride (at a concentration of 100 mm). It should be noted that a chloride salt was used instead of an acetate salt because of the interference of acetate at shorter wavelengths in CD spectroscopy. The spectra were measured at 5°C to further stabilize potential structures.

**Figure 4.**
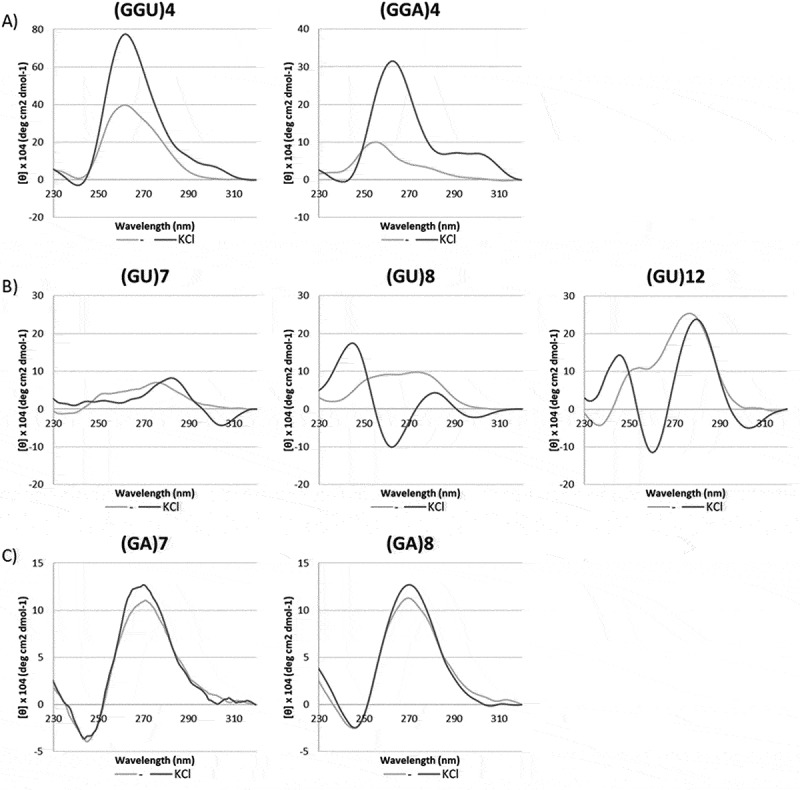
Circular dichroism spectroscopy of canonical and putative rG4 sequences. A. (GGGU)_4_, (GGU)_4_ and (GGA)_4_. B. (GGUU)_4_ and (GGAA)_4_. C. (GU)_7_, (GU)_8_, and (GU)_12_. D. (GA)_7_ and (GA)_8_. Oligonucleotides were dissolved in a 10 mm Tris buffer without (-) or with 150 mm KCl (KCl).

As references for G4 formation, we used (GGGU)_4_, (GGU)_4_ and (GGA)_4_, which are known to adopt a parallel G4 identifiable by a positive peak at 265 nm and a negative peak at 240 nm [57–60]. As shown in Figure 4(A), (GGGU)_4_ formed a G4 already in the absence of potassium chloride, whereas (GGU)_4_ and in particular (GGA)_4_ required the addition of potassium ions to fully adopt a parallel G4 structure.

The spectra of (GGAA)_4_ and (GGUU)_4_ in buffer only did not show clear features of a G4 although with (GGAA)_4_ a weak minimum at 245 nm was visible (Figure 4(B)). Addition of KCl resulted in an increase of the 265 nm peak for both RNAs but the minimum at 245 nm was absent. This suggests that these RNAs do not form a G4 structure under these conditions, however at 30°C the minimum at 245 nm was more pronounced (Supplementary Figure S5).

For the GU repeats, we obtained spectra that changed dramatically upon the addition of KCl (Figure 4(C)). The longer repeats ((GU)_8_ and (GU)_12_) showed two negative peaks at approximately at 265 and 305 nm and two positive peaks at 245 and 285 nm, when potassium was added. The spectrum of (GU)_7_ in the presence of potassium was partly similar to that of the longer repeats, with a negative peak at 305 nm and a positive peak at 285 nm, but completely lacked the maximum at 245 nm. This suggests that the peak at 245 nm is due to the presence of at least eight Gs and may be a signature of G4 formation, albeit of a type that is completely different from the G4 adopted by (GGGU)_4_, (GGU)_4_ and (GGA)_4_. (GGU)_4_, (GU)_8_ and (GU)_12_ were also measured in the presence of 100 mm LiCl. Under these conditions, the spectra largely resembled the spectra of the RNAs in buffer only indicating that the observed effects were caused by potassium ions (Supplementary Figure S6). Interestingly, when (GU)_12_ in 100 mm KCl was measured at 25°C the spectrum changed completely: the peak at 285 nm remained but those at 245 and 265 nm disappeared and a minimum at 240 nm and a maximum at 260 nm appeared (Supplementary Figure S5). This spectrum resembled that of a novel type of G4 previously reported for (GU)_12_ by Roschdi et al. [61].

The spectra of (GA)_7_ and (GA)_8_ were virtually identical and showed no shift upon addition of potassium (Figure 4(D)). Although they closely resemble the spectra of the references, their minima and maxima are shifted by ~5 nm. Besides, (GA)_7_ does not have the required number of guanines to form a G4. Therefore, it is unlikely that these GA repeats adopt a G4 unless they form a dimeric or multimeric G4 type (see Discussion).

## Discussion

In this study, we show that besides the canonical G4s made up of uninterrupted stacks of guanosines, many more G4-forming sequences potentially exist in RNA. We found that the addition of G4-specific ligands can force G-rich sequences into a putative G4 structure and that these, even for (GU)_8_ and (GU)_12_, are stable enough to halt scanning and elongating ribosomes to a similar extent as (GGGU)_4_. In addition, we found that all G-rich sequences with more than eight guanines can be forced into a G4 fold that is stable enough to affect ribosome migration, whereas translation of (GA)_7_ and (GU)_7_ were unaffected by the addition of these ligands. This same clear cut-off at seven to eight repeats is seen for both GA and GU repeats, meaning that a (GN)_8_ (or at least (GW)8 where W =A,U) repeat is sufficient for forming a functionally stable structure. Interrupted G4s have previously been found to exist in DNA, for example, *c-KIT* and *c-MYC*, whereas their presence in RNA is less well described [10,62]. The sequential addition of thymines into the well-established (GGG-N1-7-)_4_ sequence has been investigated by Mukundan & Phan who found multiple interrupted G4s in DNA that were stable [63]. Our findings suggest that G4s in RNA are capable of the same feat, provided that they are stabilized by a ligand.

The FS assay also showed that (GA)_8_ with PhenDC3 showed substantial −1 frameshifting, while the shorter (GA)_6_ showed almost no frameshifting, indicating that a stable structure can truly be formed from a minimally eighth-fold repeat on its own. In all likelihood, all G-rich sequences containing eight semi-spaced guanines would be substrates for the G4 specific ligands tested. The intrinsic stability of the G4s seems to be dependent on the length of the G-stretches, with (GGGGU)_4_ and (GGGU)_4_ showing clear −1 FSE, while (GGU)_4_ was a tenfold less effective. This corresponds with other findings reporting that two-stack G4s are innately less stable but can be stabilized by additional structural elements [64]. The addition of the stabilizing ligand PhenDC3, on the other hand, seems to result in functionally stable G4s for all these constructs. Stabilization of (GGU)_4_ even resulted in the highest FSE of 8.7%. It could be that the longer G-stretches do not always align to form three- or four-stack G4s, or that PhenDC3 does not stabilize them as effectively. It is possible for PhenDC3 to intercalate between stacks, which might result in functionally less stable G4s [65]. Another striking observation is that even the GU and GA repeats, when stabilized, reach −1 FSEs that are close to that of the uninterrupted G4 sequences. The −1 FSEs observed for the (GW)_8_ repeats and the regular G4s all reach levels that are, for example, similar to that of a frameshifting element found in HIV-1 [66]. This indicates that, after stabilization, structures formed by GW repeats could be biologically relevant.

Interestingly, while potassium and sodium ions are known to stabilize G4s, this was only observed for G4s with uninterrupted G stretches. For the constructs containing interrupted G4s, we observed that both ions do not lower TE more than they do for the negative control. This would mean that in the absence of G4 stabilizing ligands it is unlikely that GW repeats form a G4 that would be stable enough to be functionally relevant, at least not in translation. Potassium and sodium are the ions most known to stabilize G4 because the match the space between these stacks [67]. For example, Williamson et al. showed that Li^+^, which has a smaller atomic radius than K^+^ does not enhance G4 stability [68]. The added uracil interruptions in G stretches could likely open up the structure, causing a change in the ideal ion size. Thus, it would be interesting to see whether larger ions, such as Rb^+^ or Mg^2+^, can stabilize these non-canonical G4s.

**Figure 5.**
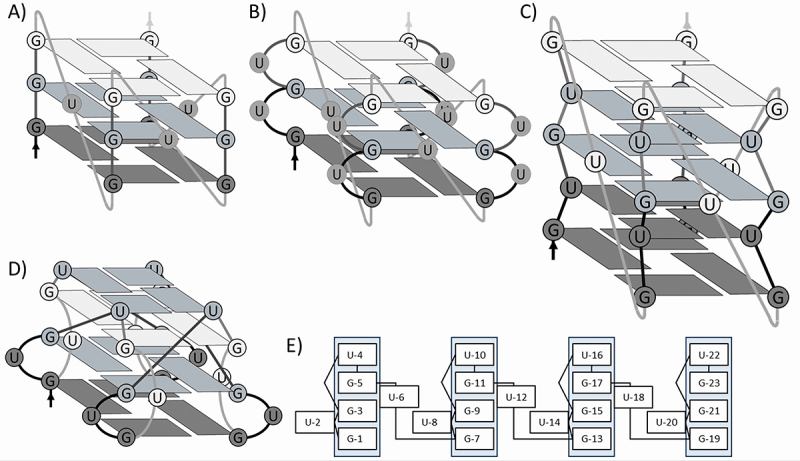
Schematic rendition of the putative G4s formed by interrupted G stretches. A) parallel G4 formed by (GGGU)_4_. B) putative G4 formed by a GU repeat where the uracils bulge out of the main structure. C) putative G4 formed by a GU repeat where the uracils form U-tetrads that stack in between the G-tetrads. We note that a single uracil may not be able to bridge the five tetrads but longer repeats can spare additional nucleotides in the loops (UGU), which might allow the formation of these kinds of G4s. D) p(UG)-fold G4, where one U-tetrad stacks on top of a G4 with bulged out uracils. E) Schematic of the pUG-Fold, where the light blue columns indicate the nucleotides that take part in forming the core of the G4 [adapted from reference [53].

Recently, Roschdi et al. proposed a novel type of G4, named the p(UG)-fold, for (GU)_12_ in the presence of potassium chloride [61]. This structure is built up from three G-tetrads and one U-tetrad (Figure 5(D,E)), which are stacked in a novel way requiring at least three G-tetrads (12 guanines). Interestingly, we did not observe a large difference in the TE of (GU)_12_ when the potassium concentration was raised, nor did we observe a difference in TE for (GU)_12_ and (GU)_8_, the latter one supposedly not capable of adopting this p(UG)-fold. Only upon addition of PhenDC3 or PDS is a structure formed that is stable enough to affect ribosome migration. It should be noted that the Tm of the p(UG)-fold G4 is 51.5°C which is substantially lower than the Tm of the canonical G4 formed by (GGGU)_4_ which lies above 85°C [57]. (GGGU)_4_, as shown here, is able to interfere with ribosomal scanning and stimulate −1 FS to a modest extent (8.5%). Consequently, the pUG-fold may simply not be stable enough to have a measurable effect on ribosome migration.

Most of the CD spectra in this work were recorded at 5°C and may not reflect the actual structures present during the translation assays. For several samples, we also measured CD at 25–30°C. Apart from changes in intensity of the signals no significant changes were observed for (GGGU)_4_, (GGAA)_4_ and (GGUU)_4_. (GU)_12_ however behaved differently at 5°C and 25°C. At 25°C the spectrum resembled the p(UG)-fold G4 as reported by Roschdi et al. [61]. At 5°C, the spectrum of (GU)_12_ (but also of GU_8_) were in line with data from Gray & Ratlif (1977) who concluded that ‘poly(rGU) self-complexed’, in other words, is forming hairpins or duplexes composed of GU base pairs [69,70]. Another possibility is that at low temperature GU repeats assume an anti-parallel G4 structure similar to telomeric repeats in the presence of NaCl [71]. Interestingly, in 100 mm NaCl, RbCl or CsCl the spectrum of (GU)_12_ is identical to that in KCl (RCLO & BM, unpublished results). It is also possible that the lower temperature at which we measured the CD spectra caused the formation of dimeric or multimeric G4s. In a recent study [72], it was shown that the CD spectrum of a (GU)_6_ repeat at high concentrations (>600 µM) resembles that of our (GU)_8_ and (GU)_12_ repeats. Remarkably at 25–100 µM (GU)_6_ was found to adopt the typical p(UG)-fold which is only possible with 12 Gs, indicating that dimerization can be an issue in CD spectroscopy (see also Basu 2024 [60]). It remains to be proven whether the p(UG)-fold is also adopted by mRNAs inside cells or whether it only forms at the high concentrations used in crystallography and CD and NMR spectroscopy.

It has been shown that the formation of rG4 can be regulated; for example, Kharel et al. showed that under stress conditions, an upregulation of rG4 occurs [73]. While fluctuations in ion concentrations are a likely cause for G4 formation in vivo, this will not affect GU/GA-based non-canonical rG4. G4 binding proteins could potentially stabilize these structures, however, causing native GU repeat G4s to exist. So far, naturally occurring ligands or G4 binding proteins stabilizing own G4 structures have only been observed in DNA however [reviewed in [74,75]]. Overall, it is our belief that these rG4 will likely not play a common role in translational regulation and might not even form readily in vivo. Whatever their structure, GW repeats are apparently targets of G4 stabilizing ligands. This has potential implications for any potential treatment that focuses on stabilizing G4s might also target these sequences. This makes the repeat sequences possible off-targets for such treatments. On the other hand, GU repeats might also be interesting to target because multiple links to GU repeat length polymorphisms and diseases have been reported [76–78]. Lastly, their likely unique fold might make it easier to target them specifically.

## Supplementary Material

Supplemental_Data_Morren_2024_revision_fin.docx

## Data Availability

The authors confirm that the data supporting the findings of this study are available within the article and its supplementary materials. Any further underlying data will be made available upon reasonable request.
