# Supplementary material for: Investigation of possible G-quadruplex formation by GU- and GA-rich repeats and their role in translation
Source: RNA Biol. 2025 Jun 2;22(1):1–12. doi: 10.1080/15476286.2025.2512613 (PMC12710887; doi:10.1080/15476286.2025.2512613)
Supplement: Supplemental_Data_Morren_2024_revision_fin.docx [file KRNB_A_2512613_SM1054.docx]

**Supplemental Data**

**Investigation of possible G-quadruplex formation by GU- and GA-rich repeats and their role in translation**

Bas M. Morren, Jitske Marcelis, Iza Muradin, René C.L. Olsthoorn

Leiden Institute of Chemistry, Leiden University, P.O. Box 9502, 2300 RA Leiden, The Netherlands

Email: [olsthoor@chem.leidenuniv.nl](mailto:olsthoor@chem.leidenuniv.nl)

Supplementary Table S1. Construct names and sequences used in luciferase assays. The sequence displayed is the sequence cloned into the pMRL vector. In bold is the repeat. Both the forward (For) and reverse (Rev) oligonucleotide sequences are shown, these were annealed prior to insertion into the digested vector.

| Name | **For/Rev** | **Sequence** |
| --- | --- | --- |
| Negative control (N.C.) | For | AAGATATAACATT**GTGGTGTGG**AATCCAAACC |
|  | Rev | TTGGATTCCACACCACAATGTTATATCTT |
| (GGGU)_4_ | For | AAGATATAACATT**GGGTGGGTGGGTGGG**AATCCAAACC |
|  | Rev | TTGGATTCCCACCCACCCACCCAATGTTATATCTT |
| (GGU)_4_ | For | AAGATATAACATT**GGTGGTGGTGGT**AATCCAAACC |
|  | Rev | TTGGATTACCACCACCACCAATGTTATATCTT |
| (GGA)_4_ | For | AAGATATAACATT**GGAGGAGGAGGA**TAATCCAAACC |
|  | Rev | TTGGATTATCCTCCTCCTCCAATGTTATATCTT |
| (GGAA)_4_ | For | AAGATATAACATT**GGAAGGAAGGAAGGAA**TAATCCAAACC |
|  | Rev | TTGGATTATTCCTTCCTTCCTTCCAATGTTATATCTT |
| (GGUU)_4_ | For | AAGATATAACATT**GGTTGGTTGGTTGGTT**AATCCAAACC |
|  | Rev | TTGGATTAACCAACCAACCAACCAATGTTATATCTT |
| (GU)_6_(GGGU)_2_ | For | AAGATATAACATT**GTGTGTGTGTGTGGGTGGG**AATCCAAACC |
|  | Rev | TTGGATTCCCACCCACACACACACACAATGTTATATCTT |
| (GUGGU)_4_ | For | AAGATATAACATT**GTGGTGTGGTGTGGTGTGG**AATCCAAACC |
|  | Rev | TTGGATTCCACACCACACCACACCACAATGTTATATCTT |
| (GU)_12_ | For | AAGATATAACATT**GTGTGTGTGTGTGTGTGTGTGTGT**AATCCAAACC |
|  | Rev | TTGGATTACACACACACACACACACACACACAATGTTATATCTT |
| (GU)_8_ | For | AAGATATAACATT**GTGTGTGTGTGTGTGT**AATCCAAACC |
|  | Rev | TTGGATTACACACACACACACACAATGTTATATCTT |
| (GU)_7_ | For | AAGATATAACATT**GTGTGTGTGTGTGT**AATCCAAACCA |
|  | Rev | TTGGATTACACACACACACACAATGTTATATCTT |
| (GA)_7_ | For | AAGATATAACATT**GAGAGAGAGAGAGA**TAATCCAAACC |
|  | Rev | TTGGATTATCTCTCTCTCTCTCAATGTTATATCTT |
| (GA)_8_ | For | AAGATATAACATT**GAGAGAGAGAGAGAGA**TAATCCAAACC |
|  | Rev | TTGGATTATCTCTCTCTCTCTCTCAATGTTATATCTT |

Supplementary Table S2. Construct names and sequences used in frameshifting assays. The sequence displayed is the sequence cloned into the pSF vector. The G-rich repeats are shown in bold. Both the forward (For) and reverse (Rev) oligonucleotide sequences are shown, these were annealed prior to insertion is a digested vector.

| Name | **For/Rev** | **Sequence** |
| --- | --- | --- |
| (GGU)_4_ | For | CTAGTA**GGTGGTGGTGGT** |
|  | Rev | CATGACCACCACCACCAA |
| (GGGU)_4_ | For | CTAGTA**GGGTGGGTGGGTGGGCA** |
|  | Rev | CATGTGCCCACCCACCCACCCTA |
| (GGGGU)_4_ | For | CTAGTA**GGGGTGGGGTGGGGTGGGGT** |
|  | Rev | CATGACCCCACCCCACCCCACCCCTA |
| (GGAA)_4_ | For | CTAGTA**GGAAGGAAGGAAGGAA** |
|  | Rev | CATGTTCCTTCCTTCCTTCCAA |
| (GGA)_4_ | For | CTAGTT**GAGGAGGAGGAGGA** |
|  | Rev | CATGTCCTCCTCCTCCAA |
| (GGA)_8_ | For | CTAGTTGA**GGAGGAGGAGGAGGAGGAGGAGGA** |
|  | Rev | CATGTCCTCCTCCTCCTCCTCCTCCTCCTCAA |
| (GA)_6_ | For | CTAGTT**GAGAGAGAGAGAAA** |
|  | Rev | CATGTTTCTCTCTCTCTCAA |
| (GA)_8_ | For | CTAGTT**GAGAGAGAGAGAGAGAG** |
|  | Rev | CATGCTCTCTCTCTCTCTCTCAA |
| (GA)_12_ | For | CTAGTT**GAGAGAGAGAGAGAGAGAGAGAGAGA** |
|  | Rev | CATGTCTCTCTCTCTCTCTCTCTCTCTCTCAA |
| (GU)_25_ | For | CTAGTT**GTGTGTGTGTGTGTGTGTGTGTGTGTGTGTGTGTGTGTGTGTGTGTGTGTGTGT** |
|  | Rev | CATGACACACACACACACACACACACACACACACACACACACACACACACACACAA |
| (GU)_8_ | For | CTAGTT**GTGTGTGTGTGTGTGTG** |
|  | Rev | CATGCTCTCTCTCTCTCTCTCAA |

Supplementary Table S3. Sequence of the 5ʹ UTR of the reference firefly luciferase control mRNA (Promega). Start codon is underlined.

| Name | **Sequence** |
| --- | --- |
| firefly luciferase control mRNA | GAAUACAAGCUUAUGCAUGCGGCCGCAUCUAGAGGGCCCGGAUCCAAAUG |


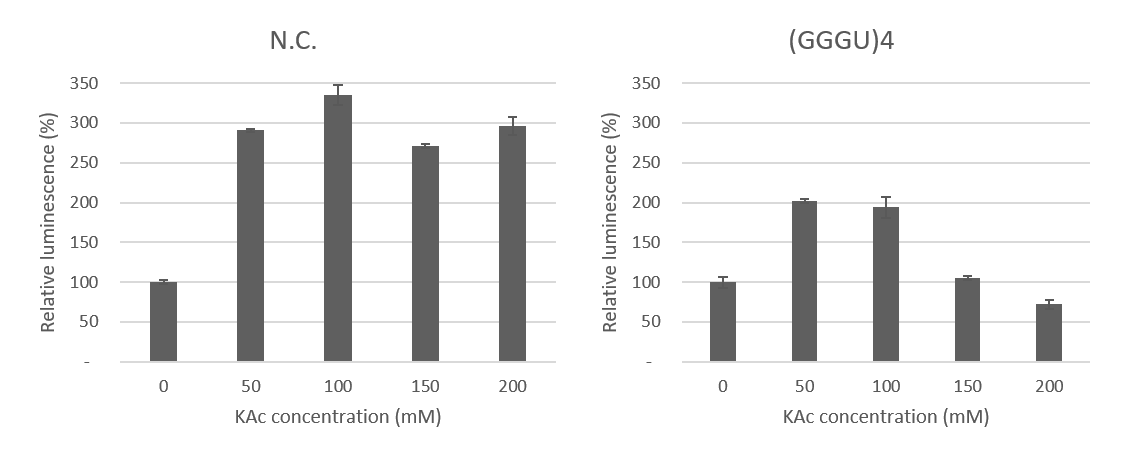


Supplementary Figure S1. Effect of potassium on translation in a 4-times diluted RRL system. Shown is the relative luminescence measured for the indicated constructs. The RRL lysate was diluted 1:1 with a buffer containing no additional potassium, before adding mRNA and increasing concentrations of potassium (see Materials & Methods) resulting in 4-times diluted RRL. Addition of potassium shows an initial increase in translation efficiency for both constructs. Above 100 mM added KAc (or ~128 mM total potassium ions) translation of the N.C. construct reaches a plateau while the (GGGU)_4_ construct shows reduced translation efficiency.


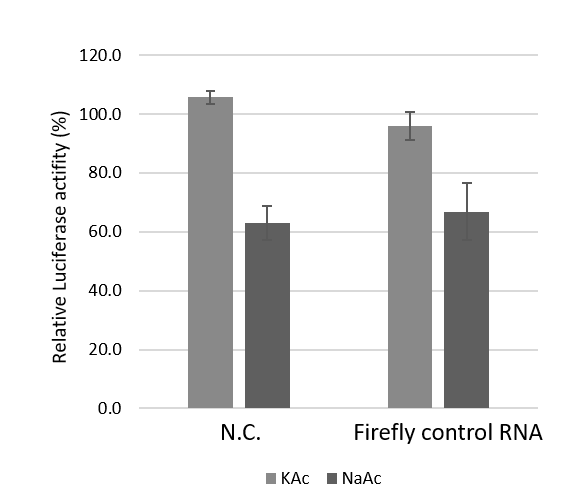


Supplementary Figure S2. Similar effects of adding KAc (150 mM) and NaAc (100 mM) on the translation of the negative control (N.C.) and a firefly control mRNA in the RRL system. The relative luminescence measured for the indicated constructs is shown. See legend to Figure 1 for further details. The firefly control mRNA is provided with the RRL lysate (Promega). See supplemental Table 3 for the 5ʹ UTR sequence.


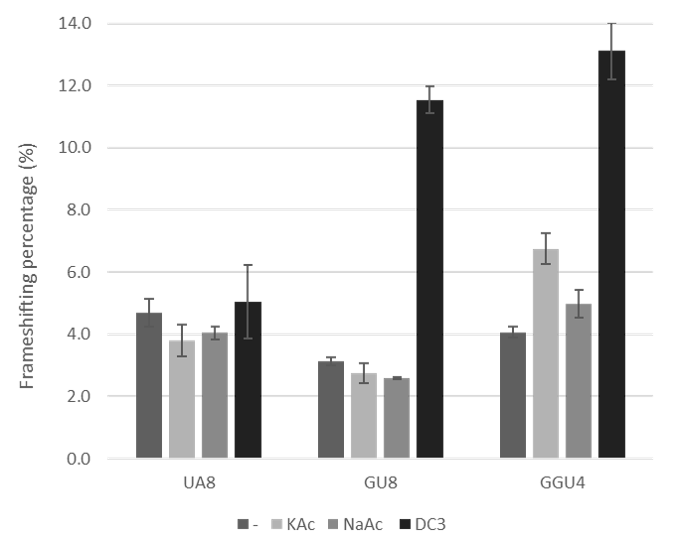


Supplementary Figure S3. Frameshift stimulation by (GU)_8_ and (GGU)_4_ measured in a dual luciferase reporter construct. The absolute frameshift efficiency is shown. See Materials & methods for further details. Potassium acetate (KAc) concentration added was 150 mM, sodium acetate (NaAc) 100 mM, PhenDC3 (DC3) 1 μM.

Supplementary Figure S4. Uncropped images of gels used to construct Figure 3.


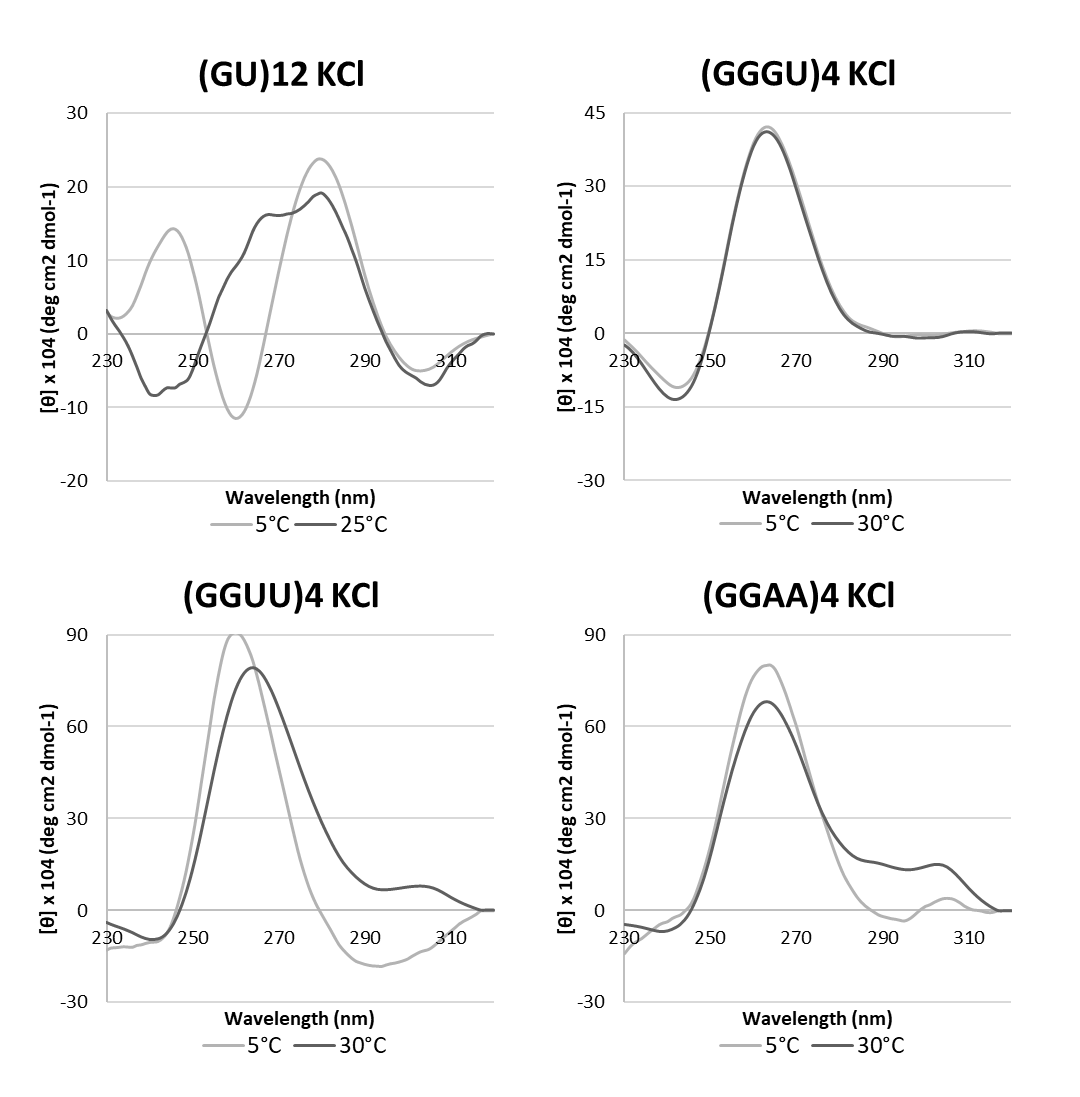


Supplementary Figure S5. Circular dichroism spectroscopy of canonical and putative rG4 sequences. (GU)_12_, (GGGU)_2_, (GGUU)_4_ and (GGAA)_4_. Oligonucleotides were dissolved in a 10 mM Tris buffer with 150 mM KCl and measured at 5 °C and at 25-30 °C.


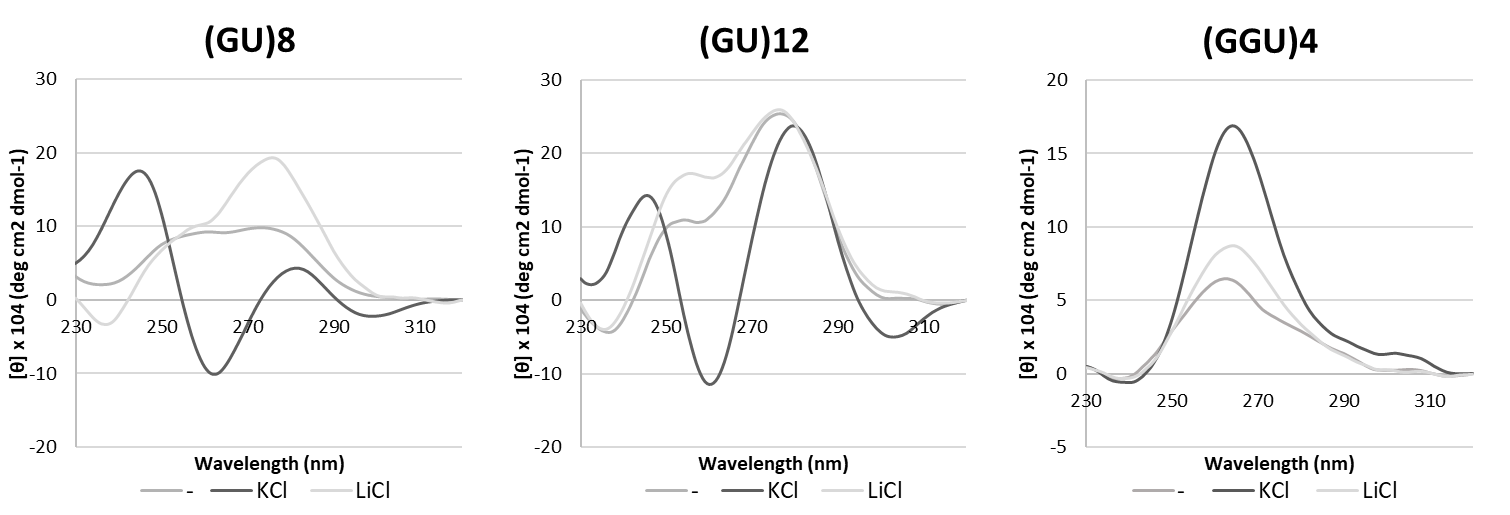


Supplementary Figure S6. Circular dichroism spectroscopy of canonical and putative rG4 sequences. (GU)_8_, (GU)_12_, and (GGU)_4_. Oligonucleotides were dissolved in a 10 mM Tris buffer without (-), with 150 mM KCl (KCl) or with 150 mM LiCl (LiCl).
